# Prevalence of Non-alcoholic Fatty Liver Disease (NAFLD) in Saudi Arabia: Systematic Review and Meta-analysis

**DOI:** 10.7759/cureus.40308

**Published:** 2023-06-12

**Authors:** Yusef M Alenezi, Rebecca Harris, Joanne Morling, Tim Card

**Affiliations:** 1 Lifespan and Population Health, University of Nottingham, Nottingham, GBR; 2 Family and Community Medicine, College of Medicine, Northern Borders University, Arar, SAU; 3 NIHR Nottingham Biomedical Research Centre (BRC), Nottingham University Hospitals NHS Trust and the University of Nottingham, Nottingham, GBR

**Keywords:** systematic review and meta analysis, non-alcoholic fatty liver, diabetes type 2, kingdom of saudi arabia (ksa), prevalence

## Abstract

Liver disease is fast emerging as a global health priority. Non-alcoholic fatty liver disease (NAFLD) is the most common liver disease in Western countries, with an increasing prevalence associated with the rising prevalence of diabetes mellitus and obesity. The worldwide prevalence of NAFLD may be in the order of 25%, but in the Middle East, it may be even higher. This study aimed to estimate the prevalence of NAFLD in the Kingdom of Saudi Arabia (KSA). A systematic review and meta-analysis were undertaken. Electronic searches were carried out through Medline, EMBASE, CINAHL, Web of Science, and Google Scholar, for articles from inception to April 2020. Studies conducted on adult populations in any setting reporting NAFLD prevalence were included. Pooled proportions and associated 95% confidence intervals (CIs) were presented in forest plots using a random effect model. Eight studies, including 4045 participants, were included. The pooled prevalence of NAFLD among all adult populations in KSA was 16.8% (11.1%-22.5%). Amongst those with type 2 diabetes, the prevalence was 58.0% (45.0%-70.9%). There were no true general population studies of the prevalence of NAFLD in KSA available. This review suggests that NAFLD is common in the KSA, and that type 2 diabetes is a risk factor in KSA as identified elsewhere in the world.

## Introduction and background

Accumulation of excessive fat in the liver is one of the common underlying mechanisms of chronic liver diseases occurring among people with non-alcoholic fatty liver disease (NAFLD) and alcohol-related liver disease (ArLD) [1-2]. This process results in a spectrum that ranges from simple steatosis through steatohepatitis, liver fibrosis, cirrhosis, and hepatocellular carcinoma (HCC) [3]. In the last three decades, NAFLD has risen to become the most common cause of chronic liver disease globally [3], mirroring the rises in obesity and metabolic syndrome [4-5]. NAFLD is prevalent worldwide, with an estimated global prevalence of 25%. However, the disease prevalence varies geographically between nations [6]. The greatest rates are reported in South America and the Middle East, followed by Asia, the United States, and Europe; NAFLD is less common in Africa. Younossi et al. estimated the prevalence of NAFLD in the Middle East as 32%, the highest globally [6-7]. The Kingdom of Saudi Arabia (KSA) is a country in the Middle East with rapid development, economic growth, and the accompanying changes in the prevalence of obesity and type 2 diabetes mellitus (T2DM). These give rise to significant health challenges and necessitate reforms in healthcare delivery [8]. In Saudi Arabia, chronic liver diseases have been historically caused by chronic viral hepatitis B (HBV) and C (HCV) more commonly than by NAFLD [9-10], with chronic viral hepatitis the leading cause of liver transplant in KSA (HCV 41.9%, HBV 21.1%) from 2001 to 2010 [11]. However, due to the control of hepatitis B by vaccination programs and the introduction of hepatitis C anti-virus therapy, the prevalence of viral hepatitis in the KSA has dramatically reduced [12-13]. In contrast, the growing epidemic of obesity, diabetes mellitus, and metabolic diseases has increased the incidence of NAFLD [14-15]. Over the last decade, non-alcoholic steatohepatitis (NASH) surpassed HBV to become the most common cause of liver transplants [11]. Furthermore, the prevalence of decompensated liver cirrhosis caused by NAFLD is projected to increase by 273% from 2017 to 2030 [16]. A significant challenge is that the prevalence of NAFLD is not well established in the KSA, limiting future planning for the disease. We aimed to undertake a systematic review and meta-analysis to determine the prevalence of this disease amongst adults in KSA to aid in the assessment of related health needs.

## Review

Materials and methods

Search Strategy

Searches were conducted by one reviewer (YA) in MEDLINE via OVID, EMBASE via OVID, CINAHL, Web of Science, and Google Scholar for articles from inception to April 2020. We searched for index terms and text words related to the concepts “prevalence of non-alcoholic fatty liver disease,” which were combined through the Boolean operator “AND” and “OR” with terms and text words related to Saudi Arabia. The search terms were as follows: [(Non-alcoholic fatty liver) OR (NAFLD) OR (Non-alcoholic steatohepatitis) OR (Non-alcoholic steatosis) OR (Steatosis) OR (Fatty liver) OR (hepatic steatosis) OR (hepatic fat)) AND ((Prevalence) or (prevalence rate) or (incidence) or (incidence rate) or (trend)) and combine search terms with ((Saudi Arabia) or (Saudia) or (Kingdom of Saudi Arabia)]. Reports, newsletters, and discussion papers were searched using the Google Internet search engine to find the gray literature in addition to the gray literature, OpenGray, and WHO library databases. Hand-searches of bibliographies from included studies and previous reviews were performed to include all possible studies that met the inclusion criteria.

Inclusion and Exclusion Criteria

We included studies investigating the prevalence of NAFLD in KSA diagnosed by any method previously validated for a diagnosis of NAFLD, such as liver biopsy, abdominal ultrasound (US), MRI, other liver scans, liver enzymes, and blood-based biomarkers -- after excluding other liver diseases [17-18]. We included both community- and hospital-based studies and those in other specific populations. Studies were excluded if the diagnosis of NAFLD was self-reported. We excluded studies of other liver diseases that cause fatty liver, including excessive alcohol consumption, side effects of certain medications (such as glucocorticoids, methotrexate, chemotherapy, and tamoxifen), and hepatitis C virus infection [19]. We included all studies that met the inclusion criteria without language or time restrictions.

Study Selection and Quality Assessment

Initially, identified articles were imported to the EndNote citation manager, and duplicates were removed. Two independent reviewers screened and assessed titles (YA and TC), abstracts (YA and JM), and then full texts (YA and JM) of the remainder. Two reviewers (YA and RH) worked independently on assessing the included studies' quality and extracting the data to a standardized form. Disagreements at all stages were resolved by consensus.

All relevant data were extracted, including the study setting classified as a hospital (inpatient or outpatient) or community. Study populations were defined as a general population when sampled from the entire population of KSA; disease-unrestricted populations when composed of people selected due to hospital/clinic attendance but not limited to a specific disease; and disease-specific populations when the cohort was selected due to the presence of a specific co-morbidity (e.g., T2DM). Diagnostic methods for NAFLD are classified as blood tests only; USS; CT; MRI; or other.

The quality of each study was evaluated based on the Joanna Briggs Institute (JBI) critical appraisal tools. The JBI critical appraisal checklist contains nine domains to assess the study's methodological quality and to determine to what extent the study has addressed the possibilities of bias in its design, conduct, and analysis. After the assessment, each domain was rated Yes, No, Unclear, or Not applicable. Studies were considered low risk and included when at least 50% of the JBI domains answered “yes” in the quality assessment checklist criteria.

Data Management and Analysis

Rayyan - Qatar Computing Research Institute (QCRI) [20] was used for article management and STATA version 16 statistical software (StataCorp LP, College Station, TX) for meta-analysis. A meta-analysis of prevalence was carried out across all included studies. We aimed to undertake subgroup analysis according to the type of population studied. Given the expectation of high heterogeneity due to the nature of the studies, a random-effects model was used to perform the meta-analysis using a weighted inverse variance model by Borenstein et al. [21]. Pooled proportions and associated 95% confidence intervals (CIs) were presented in forest plots. Higgin’s I2 test statistic was used to estimate inter-study heterogeneity, and we considered I2 > 50% to indicate high heterogeneity. p-values less than 0.05 were considered statistically significant.

Results

Search Results

**Figure 1:**
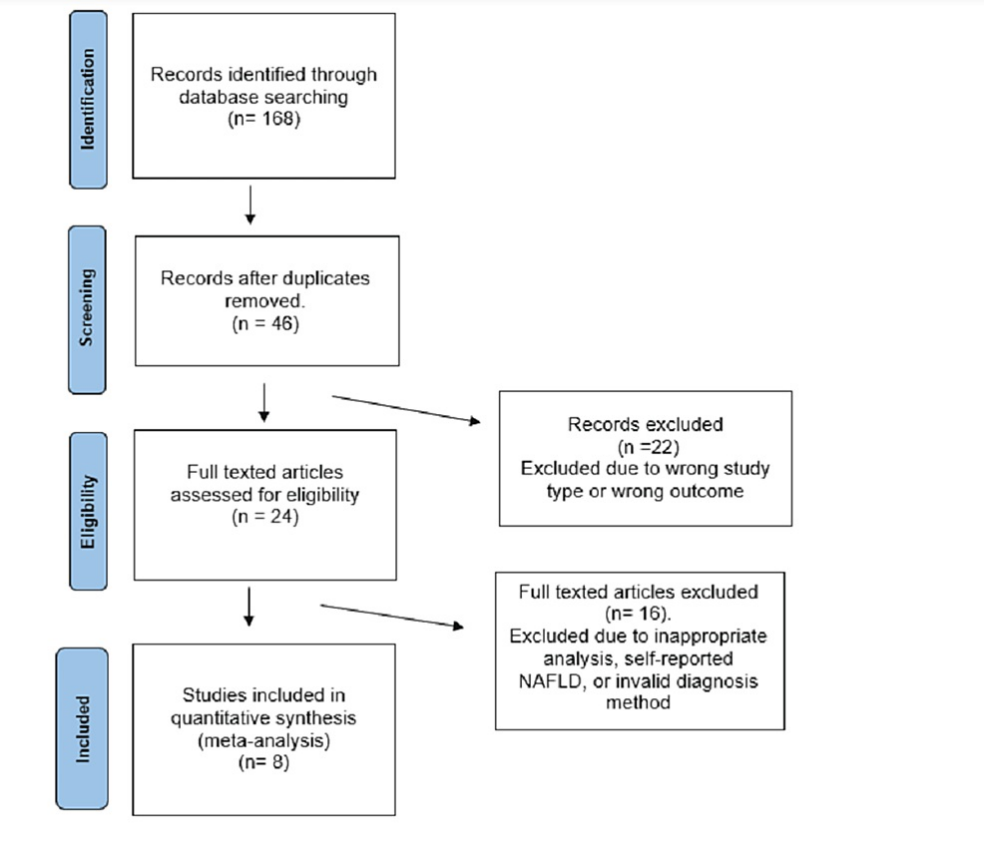
Flow diagram of articles screening and selection process for studies. Arrows represent the step-wise process. n, numbers; NAFLD, non-alcoholic fatty liver disease.

The search returned 168 publications that were included for title screening, with eight included for final data extraction (Figure 1).

NAFLD Prevalence in KSA

A total of 4045 participants from eight studies [14, 22-28] were included in the meta-analysis, of which 3405 were sampled from disease-unrestricted populations (n=4) and 640 from patients diagnosed with T2DM (n=4). Characteristics of the studies are in Table 1. Across the studies, there was a wide range of disease prevalence, from 9.7% (95% CI: 8.2-11.2) among a disease-unrestricted population in 1989 to 72.8% (95% CI: 67.1-78.6) among T2DM patients in 2017.

**Table 1:** Characteristics of included studies. Values are %(n) or mean (SD) US, ultrasound; CT, computed tomography; HU, Hounsfield unit; T2DM, type 2 diabetes mellitus; CI, confidence interval

Author Year		Study type	Study setting	Population	Participants	Males (%)	Age (years)	Diagnostic method	Prevalence (95% CI)
el-Hassan et al. [22]	1985-1989	Cohort study	Hospital-based	Disease-unrestricted population	1425	57.0	45.9 ± 15.7	CT (HU < -10)	9.7% (95% CI:8.3-11.3)
Akbar and Kawther [23]	2003	Cross-sectional	Hospital-based	T2DM patients	116	28.0	54 ± 12.8	US	55% (95% CI:46.1-63.9)
Fallatah and Akbar [24]	2008-2009	Cohort study	Hospital-based	T2DM patients	72	41.7	58.5	US	55.6% (95% CI:44.1-66.5)
Al-hamoudi et al. [14]	2009	Cross-sectional	Hospital-based	Disease-unrestricted population	1312	51.0	44.7 ± 11.5	US	16.6% (95% CI:14.7-18.7)
Alshumrani et al. [25]	2012	Cohort study	Hospital-based	Disease-unrestricted population	100	76.0	41.7 ± 11.2	CT (HU < 40)	23% (95% CI:15.8-32.2)
Elmakki et al. [26]	2013	Cross-sectional	Hospital-based	T2DM patients	207	54.1	-	US	47.8% (95% CI:41.1-54.6)
Alsabaani et al. [27]	2016	Cross-sectional	Community-based	T2DM patients	245	66.1	57.1 ± 13.5	US	72.8% (95% CI:66.6-78.1)
Alazzeh et al. [28]	2017	Cross-sectional	Community-based	Disease-unrestricted population	568	0.0	22.6 ± 4.7	US	20.4% (95% CI:17.3-23.9)

None of the studies estimated the prevalence of NAFLD in the true general population in KSA. The study most closely evaluating this was among an age-restricted (20-30 years old) sample of Saudi women, reporting a prevalence of 20.4% (95% CI:17.3-23.9).

**Figure 2:**
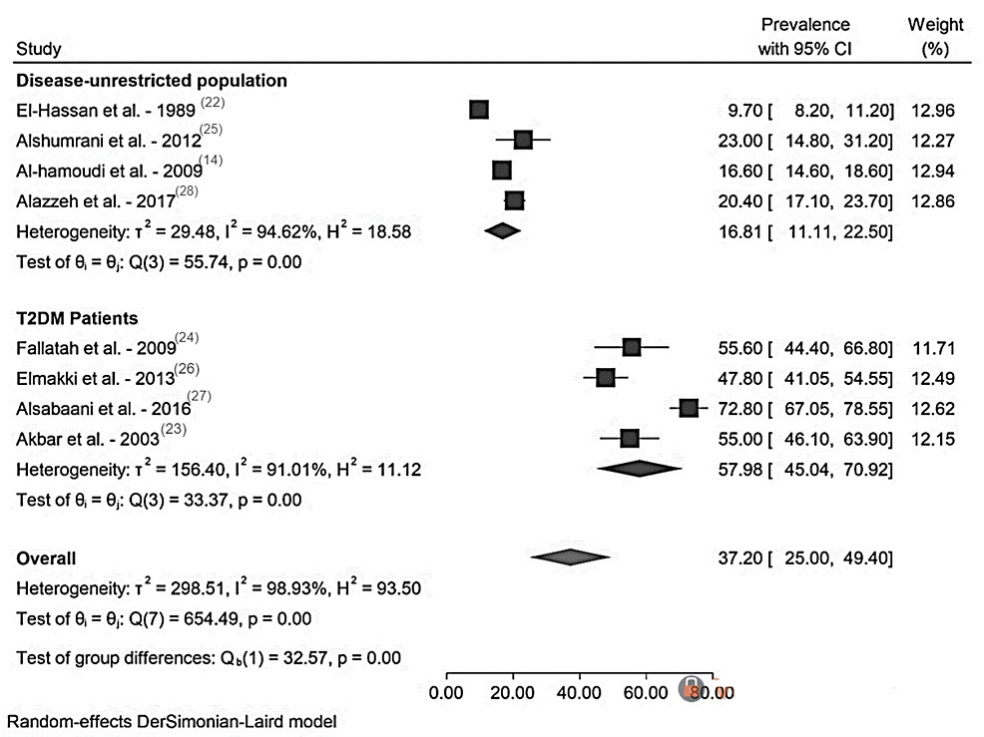
Forest plot of the prevalence of NAFLD by subgroups. Black square boxes represent the prevalence, and flat diagonally placed shape shows heterogeneity. NAFLD, non-alcoholic fatty liver disease; 95% CI, confidence interval; T2DM, type 2 diabetes mellitus

Across all studies, the pooled prevalence of NAFLD was 37.2% (95%CI: 25.0-49.6). The pooled NAFLD prevalence in the disease-unrestricted populations was 16.8% (95% CI:11.1-22.5, I2=94.6%), whereas the pooled prevalence of NAFLD among T2DM patients was 58.0% (95%CI: 45.0-70.9, I2=91.0%). Forest plots for these analyses are shown in Figure 2. The between-study variability was extremely high; for the overall analysis I2=98.9%, disease unrestricted populations I2=94.6%, and T2DM populations I2=91.0%.

Quality Assessment

A summary of the quality assessment of the included articles is presented in Table 2. All articles were of adequate quality, meeting our definition of low risk of bias. Five studies were unclear about the adequacy of sample size [14, 22-24, 28]. Sampling was not clearly explained in two studies, one inadequately described subjects and settings, and one study did not mention the response rate [23, 25, 28].

**Table 2:** Quality assessment of included studies.

Author	Was the sample frame appropriate to address the target population?	Were study participants sampled in an appropriate way?	Was the sample size adequate?	Were the study subjects and the setting described in detail?	Was the data analysis conducted with sufficient coverage of the identified sample?	Were valid methods used for the identification of the condition?	Was the condition measured in a standard, reliable way for all participants?	Was there an appropriate statistical analysis?	Was the response rate adequate, and if not, was the low response rate managed appropriately?
el-Hassan et al. [22]	Yes	Yes	Unclear	Yes	Yes	Yes	Yes	Yes	Yes
Akbar and Kawther [23]	Yes	Unclear	Unclear	Yes	Yes	Yes	Yes	Yes	Unclear
Fallatah and Akbar [24]	Yes	Yes	Unclear	Yes	Yes	Yes	No	Yes	Yes
Al-hamoudi et al. [14]	Yes	Yes	Unclear	Yes	Yes	Yes	Yes	Yes	Yes
Alshumrani et al. [25]	Yes	Yes	Yes	Unclear	Yes	Yes	No	Yes	Yes
Elmakki et al. [26]	Yes	Yes	Yes	Yes	Yes	Yes	Yes	Yes	Yes
Alsabaani et al. [27]	Yes	Yes	Yes	Yes	Yes	Yes	Yes	Yes	Yes
Alazzeh et al. [28]	Yes	Unclear	Unclear	Yes	Yes	Yes	Yes	Yes	Yes

Discussion

In this study, we found the pooled prevalence of NAFLD in KSA to be 37.2%. However, we were unable to identify any studies on the prevalence among the general population. Amongst people attending hospitals/community healthcare for any reason (disease unrestricted), the prevalence was 16.8% (95%CI 11.1-22.5), and the only disease-specific subgroup for which studies were available was T2DM with a prevalence of 58.0% (95%CI 45-70.9). The closest study to a general population was who found a prevalence of 20.4% (95% CI:17.3-23.9) in a group of young (20-30 years) women [28].

Determining the prevalence of NAFLD in the general population presents a major challenge as it involves studying asymptomatic individuals while ensuring accurate diagnostic criteria. NAFLD is often a diagnosis of exclusion and identified in a broad range of specialties and its diagnosis requires the exclusion of a number of other conditions -- as a result, there is no single diagnostic test, and there are inconsistencies in reporting. This complex diagnostic process renders the conduct of truly population-based studies complex when compared to conditions in which a single cheap diagnostic test is available. Thus, only a few such studies have been conducted. Four of the studies we included could be considered to approximate a general population study [14, 22, 25, 28]. Three of these studies included people attending for imaging (either CT or USS) for reasons unrelated to NAFLD, and the other was a cross-sectional study of female University students [14, 22, 25, 28]. Although not true general population studies, therefore, it is reasonable to consider these as providing more realistic reflections of the general population risk than is available in studies selected for reasons related to liver disease, NAFLD, or its risk factors. It is striking that the prevalence from the three disease-unrestricted imaging-based studies is close to that from the cross-sectional survey and also that these estimates are between those provided by cross-sectional surveys from Iran and Israel, which were included in the meta-analysis by Younossi et al. studying the global epidemiology of NAFLD [4, 29-30]. The overall estimate of that study which was of Middle Eastern NAFLD prevalence of 32%, was greatly raised by the inclusion of a study from Turkey conducted in gastroenterological and general internal medical outpatients, among whom 55.1% were found to have NAFLD [31]. When compared to the Younossi review, this study's estimate falls between the population-based estimates from Iran and Israel and is lower than the estimate from Turkey, which was specific to gastroenterology outpatients. Iran and Israel's estimates provide a broader representation of the disease burden in the general population, while Turkey's estimate may overestimate the disease burden due to the selection of a specific patient population.

In addition to being broadly similar to other Middle Eastern estimates of NAFLD prevalence, our results also mirror those elsewhere in showing that NAFLD is more common in diabetics. We found a pooled prevalence in studies of T2DM populations of 58.0% and this greatly elevated prevalence is reflected in studies from China, India, Africa, and Europe [32-34].

Looking forward, one-quarter of the adult population of KSA affected by T2DM is predicted to more than double by 2030 [35]. Similarly, the worrying levels of obesity in the population are projected to rise further [35]. Reflecting this, modeling estimates NAFLD prevalence of 25.7%, and with likely trends in risk factors, this has been estimated to rise by 2030 to reach 31.7% among the Saudi general population [16]. Despite the limitations of the data available, it could be argued that this rising trend is already clear within the studies we have included based on disease unrestricted imaging series. The study by el-Hassan et al. [22] utilizing CT scans from 1985 to 1989 found a NAFLD prevalence of 9.7%, which rose to 16.6% in the USS-based study of Al-hamoudi et al. in 2009 and to 23% in the CT-based study of Alshumrani in 2012 [14, 25].

This rising tide of NAFLD is likely to have a profound impact on the people and healthcare systems of KSA. NASH has already overtaken HCV hepatitis as the most common cause of liver transplant in Saudi Arabia [11]. NAFLD in general, however, progresses slowly, and so these transplants are likely to reflect NAFLD, which developed many years ago when the prevalence was lower [36]. Hence the current levels of clinical liver disease due to NAFLD are likely to be just the beginning of far larger impacts in the future. It is not yet known what the rate of progression of screen-detected NAFLD will be. We do though know that about 20% of type 2 diabetics have elevated liver stiffness internationally (suggesting the presence of significant fibrosis), that NAFLD patients with fibrosis have a high risk of progressing to cirrhosis, and that the rate of type 2 diabetes in Saudi Arabia is high and rising [36-38]. It is reasonable, therefore, to assume that there will be high levels of cirrhosis, decompensated cirrhosis, and HCC in the coming years.

The systematic methodology of this study reduces the risk of selection bias in study selection and ensures that our results are as authoritative as possible. However, as for any systematic review, the quality of the output is dependent upon the available studies. In this case, there is high heterogeneity among the studies attributable to the variety of study designs and diagnostic methods utilized. In addition, and arguably a more serious limitation, there is a paucity of studies that limits the scope and precision of the results. None of the studies addressed a true general population prevalence of NAFLD or reported on subgroups at high risk other than diabetics.

## Conclusions

Based on the currently available data, NAFLD prevalence in KSA shows that it is common and that it has similar risk factors to those elsewhere as far as can currently be assessed. It is reasonable to assume that this will become an ever-greater disease burden if obesity is not controlled and no action is taken. Without better estimates of the prevalence in both the general population and at-risk groups in KSA though, precise estimates of the size of this problem are made more difficult. Based on the available knowledge, interventions targeting obesity and T2DM in KSA are likely to be appropriate in trying to hold back this disease's tide.
